# Non-invasive evaluation of cytokine expression using the cerumen of dogs with otitis externa

**DOI:** 10.3389/fvets.2024.1355569

**Published:** 2024-02-22

**Authors:** Ji-Seon Yoon, Jinho Park

**Affiliations:** ^1^Biosafety Research Institute and Laboratory of Veterinary Deramtology, College of Veterinary Medicine, Jeonbuk National University, Iksan, Republic of Korea; ^2^Biosafety Research Institute and Laboratory of Veterinary Internal Medicine, College of Veterinary Medicine, Jeonbuk National University, Iksan, Republic of Korea

**Keywords:** dog, otitis externa, cytokine, cerumen, *Malassezia*, bacteria

## Abstract

The development of a non-invasive method to analyze cytokine expression in the skin will provide further understanding of inflammatory skin disorders. This study aimed to evaluate cytokine expression in the skin through cerumen swabbing in dogs with otitis externa (OE) and to investigate whether increased cytokine expression in infected OE reflects the inflammatory status of the ear canal. Three groups consisting of control dogs (*n* = 24), dogs with ceruminous *Malassezia* OE (*n* = 25), and dogs with suppurative bacterial OE (*n* = 15) were included in the study. The concentrations of keratinocyte-derived cytokines including Interleukin (IL)-8/chemokine ligand (CXCL)8, IL-10, IL-6, Tumor necrosis factor (TNF)-α, and IL-1ß in the cerumen of the ear canal of the included patients were analyzed using commercial ELISA kits. Additionally, correlations between cytokine levels and cytology scores (of *Malassezia* yeasts, cocci/rod-shaped bacteria, and inflammatory cells) were assessed. IL-8/CXCL8 concentrations were significantly higher in dogs with ceruminous *Malassezia* OE and dogs with suppurative bacterial OE than in control dogs. Furthermore, IL-8/CXCL8 concentrations positively correlated with *Malassezia* scores in dogs with ceruminous OE (*r* = 0.630) and with bacterial scores in dogs with suppurative OE (*r* = 0.601). In addition, increased expression of IL-6 and IL-1ß were detected in dogs with suppurative bacterial OE compared to those with *Malassezia* OE and control dogs, and showed positive correlation with inflammatory cell scores IL-6 *r* = 0.520, IL-1ß; *r* = 0.680). Therefore, keratinocyte-derived cytokines could be evaluated using non-invasive methods such as cerumen swabbing in dogs with OE.

## 1 Introduction

Cytokines are key cell-signaling proteins in various immune and homeostatic pathways and has contributed to the understanding of the pathophysiology of immune diseases in both human and veterinary medicine (1, 2). In the epidermis, keratinocytes produce a vast repertoire of cytokines, including interleukins, growth factors, colony-stimulating factors, and chemokines, in response to external stimuli such as allergens, bacterial infections, or chemical substances (3). Cytokine expression has been investigated in inflammatory skin disorders to gain insights into the physiological and pathological processes of the disease (3). Previous studies on cytokine analysis in inflammatory skin disorders have used serum cytokines or skin samples collected by biopsy (3, 4). However, the serum only reflects changes in cytokine expression throughout the body and does not reflect cytokine expression in the skin itself. Additionally, because skin biopsy is an invasive method, there are limitations in its use for collecting data from many patients. Therefore, research on non-invasive methods for analyzing skin cytokine expression has recently become of interest. These methods employ collecting samples from the cerumen, ocular surface, and stratum corneum by tape stripping (3, 5–7). These non-invasive methods allow sampling from many patients and analysis of the expression of cytokines in the skin.

Among these non-invasive methods, cerumen is a biological substance composed of lipids, proteins, amino acids, and carbohydrates produced by keratinocytes and ceruminous and reflects the pathophysiological status of the ear canal (5, 8). In addition, when inflammation occurs in the ear canal, the cerumen contains antimicrobial defense molecules, including lysozyme and immunoglobulins, as well as additional proteins with antimicrobial functions (8). A previous study investigating miRNA expression in dogs with otitis externa (OE) demonstrated miRNA profile changes between healthy and otitis-affected dogs, and bio-informatics showed that altered miRNAs in the cerumen may be involved in the modulation of the host immune response (5). In addition, a previous study using multiplex cytokine analyses in the ear canals of dogs with OE caused by atopic dermatitis showed increased levels of interleukin (IL)-8 and IL10 (6). However, the previous study selected only non-infected OE to avoid the production of pro-inflammatory cytokines related to microorganism contamination/proliferation. *Malassezia* and *Staphylococcus* spp. Stimulate cytokine production by epidermal keratinocyte (9, 10). Therefore, evaluating cytokine expression in patients with OE infected with these microorganisms provides further information of cytokine analysis using the cerumen in both primary and secondary OE infections.

The aim of this study was to determine the cytokine concentrations that can be detected using non-invasive techniques for skin surface cerumen collection. For this purpose, we selected infected OE cells that induced pro-inflammatory cytokine secretion in keratinocyte cell lines upon exposure to microbial agents. Infected OE was further divided by ceruminous *Malassezia* OE and suppurative bacterial OE and the concentration of keratinocyte-derived cytokines including IL-8/chemokine ligand (CXCL)8, IL-10, IL-6, tumor necrosis factor (TNF)-α, and IL-1ß were analyzed. In addition, to investigate whether increased cytokine expression in infected OE reflects the inflammatory status of the ear canal, cytokine concentrations were analyzed in relation to the number of infectious agents (*Malassezia* yeasts and cocci/rod-shaped bacteria) and inflammatory cells.

## 2 Materials and methods

### 2.1 Dogs

**Figure 1:**
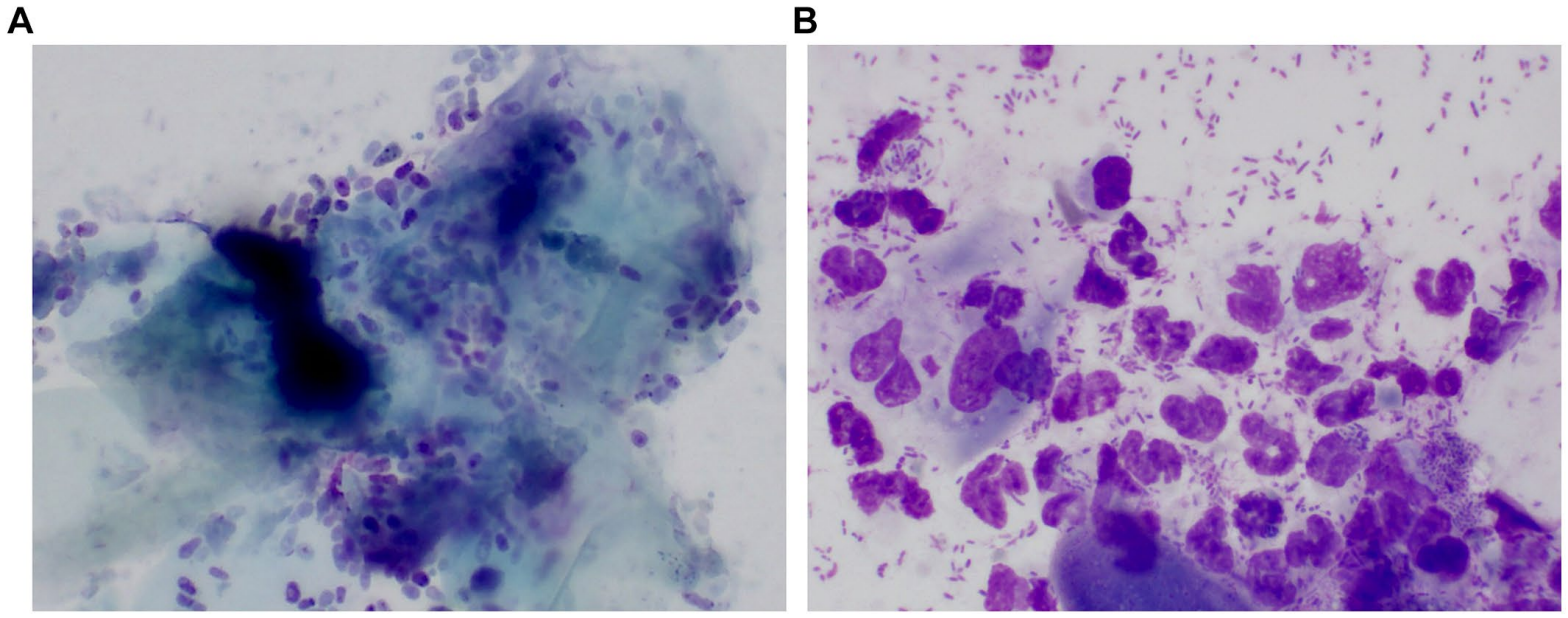
Representative pictures of cytologic examination in a dog with ceruminous *Malassezia* OE and a dog with suppurative bacteria OE The cytology of the dog with ceruminous *Malassezia* OE revealed numerous *Malassezia* yeasts and keratin were mainly observed **(A)**. One dog with suppurative bacterial OE showed large numbers of cocci/rod-shaped bacteria and inflammatory cells, such as degenerative neutrophils and macrophages. *Malassezia* was rarely observed **(B)**.

Client-owned dogs, regardless of their breed or sex, were enrolled in this study. Written informed consent from both owners and approval from the Institutional Animal Care and Use Committee of Jeonbuk National University (NON2022-101) were obtained before the study began. The dogs were divided into three groups, namely dogs with ceruminous *Malassezia* OE (*n* = 25) suppurative bacterial OE (*n* = 15), and controls (*n* = 24). Dogs with ceruminous *Malassezia* OE group had erythema and ceruminous ear discharge in the ear canal. On the cytology of ear canal swabs, *Malassezia* yeasts (more than five organisms/immersion oil field) and keratin were mainly observed (Figure 1A). The suppurative bacterial OE group included dogs that showed erosion to ulcerative lesions and purulent discharge in the ear canal. On the cytology of ear canal swabs, inflammatory cells, such as degenerative neutrophils, macrophages, and bacteria, including rods and cocci (more than 10 organisms/immersion oil field), were mainly observed, while *Malassezia* organisms were rarely seen (Figure 1B). Mixed infections with *Malassezia* yeasts and bacteria were excluded. Healthy dogs with no history of OE were included in the control group. Dogs undergoing treatment with topical or systemic antibacterial, antifungal, or anti-inflammatory therapies in the 30 days prior to the study were excluded.

### 2.2 Clinical and cytological evaluation

The ears were examined using a video-otoscope and a 0–3 Otitis Index Score (OTIS)3 was used to evaluate the severity of otitis, as previously reported (11). Erythema, hyperplasia, erosions/ulcerations, and exudates of both the vertical and horizontal ear canals were assessed on a scale of 0–3 to give a total score of 0–12. Cytology was performed by collecting the cerumen using a standard dry non-sterile cotton swab from the junction of the vertical and horizontal ear canals. The slides were dried and stained using Diff Quick staining. Cytology scores from 0 to 4 were assigned based on the presence of *Malassezia*, cocci/rod-shaped bacteria, and inflammatory cells such as neutrophils and macrophages, using modified methods of previously reported (12). Accordingly, for *Malassezia* scores in dogs with ceruminous *Malassezia* OE, a score of 0 was assigned when no round to oval yeasts of *Malassezia* were observed: 1 for 1–5, 2 for 5–10, 3 for 10–20 and 4 for >20 under immersion oil. For bacterial scores in suppurative bacteria OE, a score of 0 was given when neither cocci nor rod-shaped bacteria were observed, 1 for 1–10, 2 for 10–20, 3 for 20–40 and 4 for >40 in an immersion oil field. In addition, for the inflammatory cell score in the suppurative bacteria OE, a score of 0 was assigned where no inflammatory cells were seen: 1 for 1–5, 2 for 5–10, 3 for 10–20 and 4 for >20 within the immersion oil field. In addition, to identify bacteria agents, bacterial culture were performed in eight of 15 dogs with suppurative bacterial OE. Ear swab samples were cultured in blood agar and the colonies were identified by matrix-assisted laser desorption ionization time-of-flight (MALDI-TOF) mass spectrometry (VITEK MS v.2; bioMérieux, Inc., Durham, NC, USA).

### 2.3 Analysis of chemokine and cytokine expression in cerumen of ear canal

The cerumen was obtained by inserting dry sterile swabs into the vertical and horizontal ear canals. Ear swab samples were treated with 0.05% Tween 20 in phosphate-buffered saline for 30 min with sonication. The extracts were then centrifuged for 5 min at 2100 × g to remove skin solids, and aliquots of the extracts were collected. The aliquots were placed in dry Eppendorf tubes and frozen at −20°C until analysis. Total soluble protein concentrations were analyzed using a BCA Protein Assay Kit (Thermo Scientific, Rockford, IL, USA) with bovine serum albumin as the reference standard. Immunoassays to detect IL-8/CXCL8 (DY1608, R&D Systems, Minneapolis, MN, USA), IL-10 (DY735, R&D Systems), IL-6 (DY1609, R&D Systems), TNF-α (DY1507, R&D Systems), and IL-1ß (DY3747, R&D Systems) in the cerumen were performed according to the manufacturer’s instructions. The concentrations of canine IL-8/CXCL8, IL-10, IL-6, TNF-α and IL-1ß were calculated as pg/mg total soluble protein.

### 2.4 Statistical analysis

Statistical analyses were performed using the SPSS statistics (Version 29.0. Armonk, NY, IBM Corp.). The cytokine concentrations in the three groups were statistically analyzed using one-way analysis of variance (ANOVA), followed by Tukey’s post-hoc test. In addition, Pearson’s correlation test was used to analyze the correlation between cytokine concentrations and cytology scores. Correlation coefficients (*r*) of <−0.4 and > 0.4 indicate significant negative and positive correlations, respectively. Statistical significance was set at *p* < 0.05.

## 3 Results

### 3.1 Clinical manifestations of OE groups and control dogs

The participant’s age, sex, breed, and OTIS3 and cytology scores of dogs with *Malassezia* OE and dogs with suppurative bacterial OE are summarized in Table 1. In dogs with ceruminous *Malassezia* OE, the average OTIS3 scores were 7.60 ± 1.53, 2.04 ± 0.54, 2.28 ± 0.46, 0.92 ± 0.57, and 2.36 ± 0.46 for the total, erythema, hyperplasia, erosion/ulceration, and exudate scores, respectively. In addition, the average OTIS3 scores in dogs with suppurative bacterial OE were 9.27 ± 1.44, 2.47 ± 0.52, 1.80 ± 0.86, 2.47 ± 0.52, and 2.71 ± 0.47 for the total, erythema, hyperplasia, erosion/ulceration, and exudate scores, respectively. The average *Malassezia* cytology score in dogs with ceruminous *Malassezia* OE was 3.29 ± 0.60 and inflammatory cell score and bacterial score in dogs with suppurative bacterial OE were 2.84 ± 1.32 and 3.36 ± 1.05, respectively. In addition, cytology of suppurative bacterial OE revealed that all 15 dogs with suppurative bacterial OE showed mixed cytological infections with rods and cocci. In addition, we performed bacterial culture in eight of 15 dogs with suppurative bacterial OE, which revealed a mixed infection of gram-negative bacilli and gram-positive cocci in all eight dogs. The details of isolated bacteria are present in Supplementary Table S1.

**Table 1:** Signalments, OTIS3 scores, and cytology scores in enrolled dogs.

	Control dogs (*n* = 24)	Ceruminous *Malassezia* otitis externa (*n* = 25)		Suppurative bacterial otitis externa (*n* = 15)	
Age (years)	7.05 ± 4.32	9.07 ± 5.11		10.99 ± 3.96	
Sex	12 Castrated male	17 Castrated male		8 Castrated male	
	12 Spayed female	8 Spayed female		6 Spayed female	
				1 Female	
Breeds	6 Maltese	9 English cocker spaniel		8 English cocker spaniel	
	5 Beagle	3 Maltese		2 Maltese	
	3 Miniature poodle	3 Beagle		2 Mix	
	3 Mix	2 Mix		1 Beagle	
	2 Chihuahua	2 Miniature pincher		1 Jindo	
	1 English cocker spaniel	1 Yorkshire terrier		1 Miniature schnauzer	
	1 Miniature pincher	1 Miniature poodle			
	1 Yorkshire terrier	1 Pompitz			
	1 Pomeranian	1 Labrador retriever			
	1 Labrador retriever	1 Miniature schnauzer			
		1 Pomeranian			
Otis3 score (total 0–12)		7.60 ± 1.53		9.27 ± 1.44	
Cytology score		*Malassezia* score (0–4)	3.29 ± 0.60	Inflammatory cell score (0–4)	2.84 ± 1.32
				Bacteria score (0–4)	3.36 ± 1.05

### 3.2 Cytokine expression in cerumen of OE groups and control dogs

**Figure 2:**
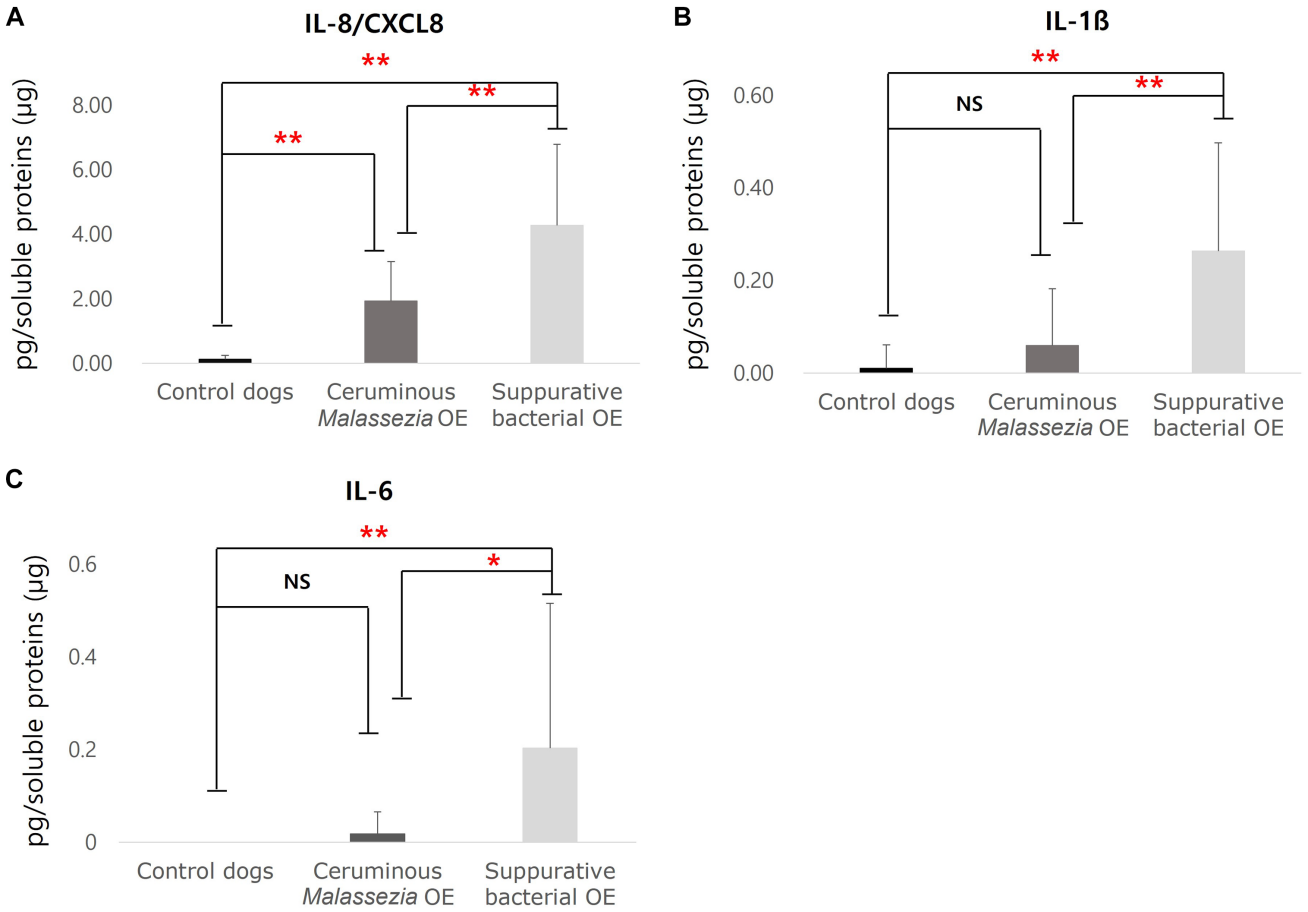
Cytokines concentrations in cerumen of control dogs, dogs with ceruminous *Malassezia* OE and dogs with suppurative bacteria OE IL-8/CXCL8 concentrations were significantly increased in ceruminous *Malassezia* OE and suppurative bacterial OE than those in control dogs, with the levels being higher in suppurative bacterial OE **(A)**. In addition, increased concentrations of IL-1ß were detected in dogs with suppurative bacterial OE, comparing those in *Malassezia* OE and control dogs **(B)**. IL-6 concentrations were significantly higher in dogs with suppurative bacterial OE than in those *Malassezia* OE and control dogs **(C)** (NS, no significant difference; **p < 0.01).

To analyze cytokine and chemokine expression in cerumen of dogs with OE, concentrations of keratinocytes-derived cyotkiens of IL-8/CXCL8, IL-10, IL-6, TNF-α, and IL-1ß were investigated using commercially available ELISA kits and calculated as pg/mg soluble protein. In both groups of ceruminous *Malassezia* OE (*p* < 0.01) and suppurative bacterial OE (*p* < 0.01), IL-8/CXCL8 concentrations were significantly higher than those in the control dogs (Figure 2A). Furthermore, IL-8/CXCL8 concentrations in subjects with suppurative bacterial OE were significantly higher than those in subjects with ceruminous *Malassezia* OE (*p* < 0.01, Figure 2A). In addition, increased expression of IL-1ß was detected in dogs with suppurative bacterial OE, compared to those with *Malassezia* OE and control dogs (*p* < 0.01, Figure 2B). IL-6 concentrations were significantly higher in dogs with suppurative bacterial OE than in those *Malassezia* OE and control dogs (*p* < 0.01, Figure 2C). In contrast, in *Malassezia* OE and control dogs, IL6 was barely detected using the immunoassay used in this study (Figure 2C). In addition, expression of IL-10 and TNF-α were undetectable in all subjects using commercially available immunoassay kit used in this study.

### 3.3 Correlation of cytokines concentrations with cytology scores

**Figure 3:**
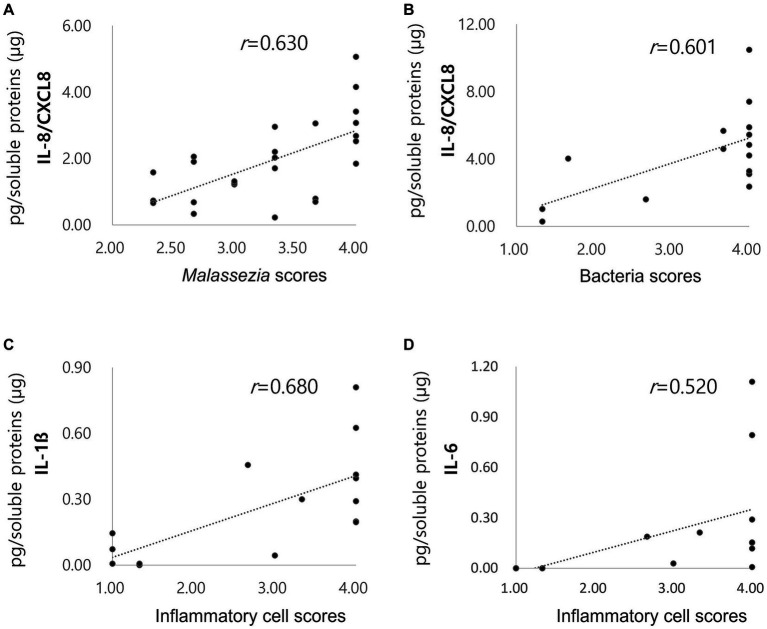
Correlations of cytokine concentrations with *Malassezia* score, bacteria score and inflammatory score. Increased concentrations of acute phase proteins in dogs with pyometra The IL-8/CXCL8 concentration in ceruminous *Malassezia* OE positively correlated with *Malassezia* scores (**(A)**, *r* = 0.630, *p* < 0.01). In suppurative bacterial OE, the IL-8/CXCL8 concentration positively correlated with the bacterial scores (**(B)**, *r* = 0.601, *p* = 0.018). IL-6 **(C)** and IL-1ß **(D)** concentrations showed positive correlation with inflammatory cell scores (IL6; *r* = 0.520, *p* = 0.047, IL-1ß; *r* = 0.680, *p* < 0.01).

The correlation of *Malassezia* score with IL-8/CXCL8 concentrations in dogs with ceruminous *Malassezia* OE, and the correlation of inflammatory cell scores and bacterial scores with IL-8/CXCL8, IL-6, and IL-1ß concentration in dogs with suppurative bacterial OE were investigated. In dogs with ceruminous *Malassezia* OE, IL-8/CXCL8 concentrations showed positive correlation with *Malassezia* score (Figure 3A, *r* = 0.630, *p* < 0.01). In dogs with suppurative bacterial OE, the IL-8/CXCL8 concentration showed a positive correlation with the bacterial score (Figure 3B, *r* = 0.601, *p* = 0.018), whereas no significant correlation was noted with the neutrophil score (data not shown). Additionally, IL-6 and IL-1ß concentrations showed positive correlation with inflammatory cell scores (Figures 3C,D, IL6; *r* = 0.520, *p* = 0.047, IL-1ß; *r* = 0.680, *p* < 0.01). However, no significant correlations were observed between bacteria scores and IL-6 and IL-1ß concentrations (data not shown).

## 4 Discussion

Cerumen analysis is a non-invasive method that is able to reflect the inflammatory status of dogs (8). The present study evaluated skin cytokine expression in dogs infected with OE using non-invasive cerumen swabbing methods. Cytokines in the cerumen are differentially expressed between ceruminous *Malassezia* OE and suppurative bacterial OE. Accordingly, ceruminous *Malassezia* OE showed increased IL-8/CXCL8 concentrations than those of the control, while suppurative bacterial OE showed significantly increased levels of IL-6, IL-1ß, and IL-8/CXCL8. IL-8/CXCL8 levels are higher in suppurative bacterial OE than in ceruminous *Malassezia* OE. Furthermore, increased cytokine levels positively correlated with the number of infectious agents or inflammatory cells. The present study indicates that non-invasive methods of cerumen swabbing in dogs with OE allow the analysis of cytokine expression, which might reflect the pathophysiological status of the ear canal. Keratinocyte-derived cytokines may be differentially expressed depending on the presence or absence of pathogens or inflammatory cells.

IL-8/CXCL8 is a member of the supergene family of pro-inflammatory and chemotactic cytokines, and is produced by macrophages and other cell types, such as the epithelial cells of keratinocytes (13). In this study, both ceruminous *Malassezia* OE and suppurative bacterial OE showed significantly increased IL-8/CXCL8 concentrations. Furthermore, IL-8/CXCL8 concentrations were positively correlated with *Malassezia* infectious organisms and bacteria. Similar findings have been reported with IL-8 concentrations in the ear canal of ceruminous in dogs with OE were significantly decreased with the reduction of *Malassezia* organisms due to the use of ear cleaners (12). Previous studies have shown that *Malassezia* species induce IL-8 secretion by human keratinocytes (9, 14-16). Therefore, the increased IL-8/CXCL8 concentrations in ceruminous *Malassezia* OE may be related to the high number of *Malassezia* organisms that induce IL-8/CXCL8 secretion from epidermal keratinocytes. In addition, it has been reported that bacteria such as *Staphylococcus* spps. Directly induce IL-8 production in human keratinocytes (10). In this study, the increased IL-8/CXCL8 concentrations in suppurative bacterial OE might be associated with increased bacterial colonization in the ear canal. IL-8 concentrations were significantly higher in dogs with suppurative bacterial OE than in those with ceruminous *Malassezia* OE. These results were due to higher numbers of bacteria in suppurative OE than *Malassezia* yeast in ceruminous OE and/or the presence of other inflammatory cells in suppurative OE.

Similarly, significantly increased IL-8/CXCL8 concentrations have been reported in previous studies that analyzed cytokines by swabbing the cerumen or occlusal surface of dogs with atopic dermatitis (6, 7). In an analysis of cytokine expression in conjunctival swabs, IL-8 was the only cytokine that was significantly increased in atopic dogs, showing a positive correlation with conjunctival and pruritus scores (7). In addition, the concentration of IL-8 is also significantly higher in the ears of atopic dogs than in healthy dogs (6). Interestingly, in contrast to the present study, the previous study only selected non-infected otitis patients to avoid the production of pro-inflammatory cytokines related to microbes. As IL-8/CXCL8 concentrations are affected by infectious organisms, including *Malassezia* and bacterial agents, infectious organisms should be excluded when studying IL-8 as a biomarker of atopic dermatitis.

IL-1β and IL-6 are pro-inflammatory cytokines that are crucial for host-defense responses to infection and injury. They are produced and secreted by various cell types, particularly those of the innate immune system, such as monocytes and macrophages (17, 18). In addition, it has been reported that *Malassezia* yeasts and bacteria stimulate IL-1β and IL-6 production from epidermal keratinocytes (10, 19–21). However, in this study, no significant increases in these cytokines were observed in the dogs with ceruminous *Malassezia* OE; specifically, IL-6 was barely detected in these samples. A previous study using the Luminex® assay demonstrated that IL-6 was undetectable in the ear canal of dogs with atopic OE (6). In contrast, this study showed significantly increased concentrations of IL-1ß and IL-6 in the cerumen of dogs with suppurative bacterial OE. In addition, the concentrations of these cytokines were positively correlated with inflammatory cell scores. Therefore, it is possible that in the case of OE with purulent inflammation, the expression of IL6 is increased to recruit neutrophils and/or to further increase cytokine production by inflammatory cells. Therefore, when studying cytokines through skin surface swabbing, IL-1ß and IL-6 can be selected as targets when purulent inflammation is present.

The expressions of IL-10 and TNF-α was not detected on the ELISA essay used on all subjects. In a previous study using Luminex® technology, IL-10 concentration was significantly increased in the ear canal of dogs with atopic OE (6). The assay range of the IL-10 ELISA kits used in this study was 31.2 to 2,000 pg/mL,while predefined kits using Luminex® technology in the previous study had a range of 12 to 50,000 pg/mL (6). Therefore, the lack of detectable IL-10 in this study might be related to the analytical method in which the commercially available ELISA kits used in this study has a relatively lower sensitivity for IL-10 in the cerumen of dog ear canals. Additionally, since TNF-α was not detectable in a study using Luminex® assay (6), TNF-α might be minimally expressed in cerumen.

The potential limitations of this study are as follows. (1) Because the primary factors for OE have not been fully identified, the effects of primary factor on cytokine expressions have not been completely ruled out. Primary factor of OE such as allegic skin disease and eondocrinopaty could affect cytokine expressions. There might be cytokines that could not be detected because the cytokine analysis was performed using commercially available ELISA kits. Large-scale studies of several types of OE using other assays such as the Luminex® assay are required to further understand cytokine expression as biomarkers in dogs with OE.

In conclusion, the present study demonstrated that increased IL-8/CXCL8, IL-6, and IL-1ß levels were detected using non-invasive cerumen swabbing in dogs with OE infected with *Malassezia* and bacteria. The present study provides fundamental data for future analysis of skin cytokine expression, especially using non-invasive methods such as swabbing the cerumen, ocular surface, and tape stripping of the stratum corneum. Target cytokines can be eliminated depending on the presence or absence of infectious agents and inflammatory cells.

## Data availability statement

The raw data supporting the conclusions of this article will be made available by the authors, without undue reservation.

## Ethics statement

The animal studies were approved by Institutional Animal Care and Use Committee of Jeonbuk National University. The studies were conducted in accordance with the local legislation and institutional requirements. Written informed consent was obtained from the owners for the participation of their animals in this study.

## Author contributions

J-SY: Writing – original draft, Investigation. JP: Conceptualization, Writing – review & editing.

## References

[1] Liu TJ Lin LL McMeniman E Wu J Kao YC Kumari S. Cytokine/chemokine assessment as a complementary diagnostic tool for inflammatory skin diseases. Front Immunol. (2022) 13:1028435. doi: 10.3389/fimmu.2022.1028435, PMID: 36466878 PMC9709404

[2] Tamamoto-Mochizuki C Santoro D Saridomikelakis MN Eisenschenk MNC Hensel P Pucheu-Haston C. International committee on allergic diseases of animals (ICADA). Update on the role of cytokines and chemokines in canine atopic dermatitis. Vet Dermatol. (2023) 35:25–39. doi: 10.1111/vde.13192, PMID: 37485553

[3] Portugal-Cohen M Kohen R. Non-invasive evaluation of skin cytokines secretion: an innovative complementary method for monitoring skin disorders. Methods. (2013) 61:63–8. doi: 10.1016/j.ymeth.2012.10.002, PMID: 23063704

[4] Fedenko ES Elisyutina OG Filimonova TM Boldyreva MN Burmenskaya OV Rebrova OY. Cytokine gene expression in the skin and peripheral blood of atopic dermatitis patients and healthy individuals. Self Nonself. (2011) 2:120–4. doi: 10.4161/self.2.2.16939, PMID: 22299064 PMC3268998

[5] Lecchi C Zamarian V Borriello G Galiero G Grilli G Caniatti M. Identification of altered miRNAs in cerumen of dogs affected by otitis externa. Front Immunol. (2020) 11:914. doi: 10.3389/fimmu.2020.00914, PMID: 32547539 PMC7273745

[6] Lecru LA Combarros D Moog F Marinovic L Kondratjeva J Amalric N. Multiplex cytokine analyses in ear canals of dogs suggest involvement of IL-8 chemokine in atopic otitis and Otodectic mange-preliminary results. Animals. (2022) 12:575. doi: 10.3390/ani12050575, PMID: 35268144 PMC8909880

[7] Pressanti C Ravailhe E Castellote-Brun J Amalric N Lecru LA Kondratjeva J. Survey of cytokines on ocular surfaces of atopic dogs by multiplex analysis using two sampling methods -a pilot study. Vet Dermatol. (2021) 32:625–e167. doi: 10.1111/vde.13010, PMID: 34390059

[8] Shokry E Filho NRA. Insights into cerumen and application in diagnostics: past, present and future prospective. Biochem Med. (2017) 27:030503. doi: 10.11613/BM.2017.030503, PMID: 29180914 PMC5696747

[9] Sparber F LeibundGut-Landmann S. Host responses to *Malassezia* spp. in the mammalian skin. Front Immunol. (2017) 8:1614. doi: 10.3389/fimmu.2017.01614, PMID: 29213272 PMC5702624

[10] Sasaki T Kano R Sato H Nakamura Y Watanabe S Hasegawa A. Effects of staphylococci on cytokine production from human keratinocytes. Br J Dermatol. (2003) 148:46–50. doi: 10.1046/j.1365-2133.2003.05017.x, PMID: 12534593

[11] Nuttall T Bensignor E. A pilot study to develop an objective clinical score for canine otitis externa. Vet Dermatol. (2014) 25:530–7. doi: 10.1111/vde.12163, PMID: 25130194

[12] King SB Doucette KP Seewald W Forster SL. A randomized, controlled, single-blinded, multicenter evaluation of the efficacy and safety of a once weekly two dose otic gel containing florfenicol, terbinafine and betamethasone administered for the treatment of canine otitis externa. BMC Vet Res. (2018) 14:307. doi: 10.1186/s12917-018-1627-5, PMID: 30305092 PMC6180657

[13] Kondo S Kono T Sauder DN McKenzie RC. IL-8 gene expression and production in human keratinocytes and their modulation by UVB. J Invest Dermatol. (1993) 101:690–4. doi: 10.1111/1523-1747.ep12371677, PMID: 8228330

[14] Moog F Mivielle J Dumitrache MO Amalric N Lecru LA. Clinical and Microbiological Performances and Effects on Lipid and Cytokine Production of a Ceruminolytic Ear Cleaner in Canine Erythemato-Ceruminous Otitis Externa. Vet Sci. (2022) 9:185. doi: 10.3390/vetsci9040185, PMID: 35448682 PMC9031221

[15] Buommino E De Filippis A Parisi A Nizza S Martano M Iovane G. Innate immune response in human keratinocytes infected by a feline isolate of *Malassezia pachydermatis*. Vet Microbiol. (2013) 163:90–6. doi: 10.1016/j.vetmic.2012.12.001, PMID: 23273837

[16] Park HR Oh JH Lee YJ Park SH Lee YW Lee S. Inflammasome-mediated inflammation by *Malassezia* in human keratinocytes: a comparative analysis with different strains. Mycoses. (2021) 64:292–9. doi: 10.1111/myc.13214, PMID: 33206994

[17] Lopez-Castejon G Brough D. Understanding the mechanism of IL-1β secretion. Cytokine Growth Factor Rev. (2011) 22:189–95. doi: 10.1016/j.cytogfr.2011.10.001, PMID: 22019906 PMC3714593

[18] Tanaka T Narazaki M Kishimoto T. IL-6 in inflammation, immunity, and disease. Cold Spring Harb Perspect Biol. (2014) 6:a016295. doi: 10.1101/cshperspect.a016295, PMID: 25190079 PMC4176007

[19] Zhang YJ Han Y Sun YZ Jiang HH Liu M Qi RQ. Extracellular vesicles derived from *Malassezia furfur* stimulate IL-6 production in keratinocytes as demonstrated in *in vitro* and *in vivo* models. J Dermatol Sci. (2019) 93:168–75. doi: 10.1016/j.jdermsci.2019.03.001, PMID: 30904352

[20] Watanabe S Kano R Sato H Nakamura Y Hasegawa A. The effects of *Malassezia* yeasts on cytokine production by human keratinocytes. J Invest Dermatol. (2001) 116:769–73. doi: 10.1046/j.1523-1747.2001.01321.x, PMID: 11348468

[21] Brauweiler AM Goleva E Leung DYM. *Staphylococcus aureus* Lipoteichoic acid damages the skin barrier through an IL-1-mediated pathway. J Invest Dermatol. (2019) 139:1753–61.e4. doi: 10.1016/j.jid.2019.02.006, PMID: 30779913 PMC6650368

