# Supplementary material for: Non-invasive evaluation of cytokine expression using the cerumen of dogs with otitis externa
Source: Front Vet Sci. 2024 Feb 22;11:1355569. doi: 10.3389/fvets.2024.1355569 (PMC10921565; doi:10.3389/fvets.2024.1355569)
Supplement: Supplementary file 1 [file Table_1.DOCX]

Supplemental Table 1. Bacteria identified in cerumen swabs from eight dogs with suppurative bacterial otitis externa

| dogs | Identified bacteria |  | |  |
| --- | --- | --- | --- | --- |
| No.1 | *Proteus mirabilis* | *Streptococcus canis* |  | |
| No.2 | *Proteus mirabilis* | *Streptococcus canis* | *Arcanobacterium spp.* | |
| No.3 | *Proteus mirabilis* | *Streptococcus canis* | *Enterococcus faecalis* | |
| No.4 | *Proteus mirabilis* | *Staphylococcus pseudintermedius* | *Streptococcus equi* | |
| No.5 | *Proteus mirabilis* | *Streptococcus canis* | *Enterococcs faecalis* | |
| No.6 | unidentified gram-negative bacilli | *Staphylococcus pseudintermedius* |  | |
| No.7 | *Pseudomonas aeruginosa* | *Staphylococcus lutrae* | *Enterococcus faecalis* | |
| No.8 | *Proteus mirabilis* | *Streptococcus agalactiae* |  | |
| No.9 | *Pseudomonas aeruginosa.* | *Staphylococcus pseudintermedius* | *Corynebacterium species.* | |
